# Co-Crystals: A Novel Approach to Modify Physicochemical Properties of Active Pharmaceutical Ingredients

**DOI:** 10.4103/0250-474X.57283

**Published:** 2009

**Authors:** A. V. Yadav, A. S. Shete, A. P. Dabke, P. V. Kulkarni, S. S. Sakhare

**Affiliations:** Krishna Institute of Medical Scinces University and Institute of Pharmacy, Karad-415 110, India; 1 Shree Santkrupa College of Pharmacy, Ghogoan, Karad-415 111, India; 2 Goverenment College of Pharmacy, Vidyanagar, Karad-415 124, India; 3 Government College of Pharmacy, Osmanpura, Aurangabad-431 005, India; 4 Gourishankar Education Society's Institute of Pharmaceutical Sciences and Research, Limb, Satara-415 004, India

**Keywords:** Crystal form, bioavailability, drug solubility, dissolution, physicochemical property

## Abstract

Crystal form can be crucial to the performance of a dosage form. This is especially true for compounds that have intrinsic barriers to drug delivery, such as low aqueous solubility, slow dissolution in gastrointestinal media, low permeability and first-pass metabolism. The nature of the physical form and formulation tends to exhibit the greatest effect on bioavailability parameters of water insoluble compounds that need to be given orally in high doses. An alternative approach available for the enhancement of drug solubility, dissolution and bioavailability is through the application of crystal engineering of co-crystals. The physicochemical properties of the active pharmaceutical ingredients and the bulk material properties can be modified, whilst maintaining the intrinsic activity of the drug molecule. This article covers the advantages of co-crystals over salts, solvates (hydrates), solid dispersions and polymorphs, mechanism of formation of co-crystals, methods of preparation of co-crystals and application of co-crystals to modify physicochemical characteristics of active pharmaceutical ingredients along with the case studies. The intellectual property implications of creating co-crystals are also highly relevant.

Chemists and engineers in the pharmaceutical industry generally seek to deliver crystalline forms of their active compounds, mainly due to the inherent stability of crystalline materials and the well-established impact of crystallization processes on purification and isolation of chemical substances[1]. Increasing attention is now being paid to the impact of material properties on drug discovery and early development[2] as the drug substances tend to be very valuable materials. The pharmaceutical industry's mission is to rapidly advance development programs with good confidence so that formulation problems are unlikely to arise and to maximize a compounds potential as a therapeutic. This commentary seeks to raise the profile of crystal form studies and the emerging topic of crystal engineering in pharmaceutical science by discussing the state-of-the-art relating to pharmaceutical crystals and by expanding on possibilities that exist for future developments. In keeping with the common goal of making better products, faster and cheaper, we propose a paradigm of pro-active material design in pharmaceutical research. Rather than settling for the physical forms that the pure compounds intrinsically display, we should be aiming to identify the physical properties required of the target drug and make crystalline forms to meet those needs. We are not yet actively engaging in crystal engineering in the industry, and therefore we pose the question, how can we proceed toward making the crystal form that we want to use?

Pharmaceutical active ingredients (APIs) can exist in a variety of distinct solid forms, including polymorphs, solvates, hydrates, salts, co-crystals and amorphous solids. Each form displays unique physicochemical properties that can profoundly influence the bioavailability, manufacturability purification, stability and other performance characteristics of the drugs[3].

Solid form discovery and design depends on the nature of the molecule of interest and type of physical property challenges faced in its development. The preferred solid form is generally the thermodynamically most stable crystalline form of the compound[3,4]. However, the stable crystal form of the parent compound may exhibit inadequate solubility or dissolution rate resulting in poor oral absorption, particularly for water-insoluble compounds. In this case, alternative solid forms may be investigated. For ionizable compounds, preparation of salt forms using pharmaceutically acceptable acids and bases is a common strategy to improve bioavailability[3,5,6]. Like the parent compound, pharmaceutical salts may exist in several polymorphic, solvated and/or hydrated forms.

Crystal engineering is generally considered to be the design and growth of crystalline molecular solids with the aim of impacting material properties. A principal tool is the hydrogen bond, which is responsible for the majority of directed intermolecular interactions in molecular solids. Co-crystals are multi-component crystals based on hydrogen bonding interactions without the transfer of hydrogen ions to form salts; this is an important feature, since Bronsted acid-base chemistry is not a requirement for the formation of a co-crystal. Co-crystallization is a manifestation of directed self-assembly of different components. Co-crystals have been described of various organic substances over the years[7,8] and given various names, such as addition compounds[9,10] molecular complexes[11,12] and heteromolecular co-crystals[13]. Regardless of naming convention, the essential meaning is that of a multi-component crystal where no covalent chemical modification of the constituents occurs as a result of the crystal formation.

Pharmaceutical co-crystals can be defined as crystalline materials comprised of an API and one or more unique co-crystal formers, which are solids at room temperature.

Co-crystals can be constructed through several types of interaction, including hydrogen bonding, p stacking, and vander Waals forces. Solvates and hydrates of the API are not considered to be co-crystals by this definition. However, co-crystals may include one or more solvent/water molecules in the crystal lattice[14]. Co-crystals often rely on hydrogen-bonded assemblies between neutral molecules of API and other component. For nonionizable compounds co-crystals enhance pharmaceutical properties by modification of chemical stability, moisture uptake, mechanical behaviour, solubility, dissolution rate and bioavailability[15].

## SALTS, SOLVATES (HYDRATES), SOLID DISPERSIONS, POLYMORPHS AND CO-CRYSTALS

Pharmaceutical co-crystallization, which has only recently gained widespread attention as a means of modifying the physicochemical properties of APIs, has two inherent advantages over the salt form. First, because co- crystal formation may potentially be employed with all APIs, including acidic, basic and non ionisable molecules and second is a large number of potential ‘counter molecules’ which may be considered to be non toxic possibly increasing the scope of the pharmaceutical co- crystallization over the salt forms.

An analogy can be drawn to salt selection in which pKa arguments are used to select acid-base pairs that can be converted to salt compounds. Chemistry demonstrates that a pKa difference of at least two units (between an acid and a base) is required to form a salt that is stable in water[16].

It is also important to remember that salt formation is generally directed at a single acidic and basic functional group. In contrast co-crystals can simultaneously address multiple functional groups in a single drug molecule. In addition space is not limited to binary combinations (acid-base pairs) since tertiary and quaternary co-crystals are realistic one[17,18]. One very interesting thing was observed that co-crystals provide a powerful means to tailor the desired solubility and dissolution-pH dependence of APIs, even when the API is a non-ionizable molecule[19].

The extent of polymorphism of pharmaceutical is limited to the handful of the different crystal forms. Primary difference between solvates and co-crystals is the physical state of the individual components[20]. If one component is liquid at room temperature then the crystals are designated solvates, whereas if both components are solids at room temperature then the crystals are designated as co-crystals. Solvates are commonplace because they occur as a serendipitous result of crystallization from solution[21] and have the potential to enhance drug dissolution rate, as shown for the solvated forms of spiranolactone[22]. Solvated crystals however are often unstable, leading to desolvation during storage and such solvent loss may lead to the amorphous phase crystallizing into less soluble forms. Solvent levels in solvated crystals are also often at concentrations that are not acceptable to regulatory authorities and which may also have toxicological consequences. Co-crystals, however, tend to be a product of more rational design and are more stable, particularly as the co-crystallizing agents are solids at room temperature. As with other crystalline systems, polymorphic co-crystals are not uncommon. At least 20 have been reported to date, including caffeine and glutaric acid polymorphic co-crystals[23] whilst co-crystals are defined by a single phase (miscible) multi-component system in the crystalline state, in the amorphous state they have been referred to as molecular dispersions[24,25] with interactions between the components distinguishing them from solid dispersions. Co-crystals are not classified as solid dispersions; nevertheless solid dispersions may occur when attempting to prepare co-crystals from solution. The generic term solid dispersions refers to the dispersion of one or more active ingredients in an inert carrier in a solid state, frequently prepared by the melting (fusion) method, solvent method, or fusion solvent-method[26,27]. The solid dispersion approach to reduce particle size and therefore increase the dissolution rate and absorption of drugs was first recognized in 1961[26]. In the preparation of solid dispersions, drugs with a poor ability to form the glassy state, and which demonstrate notable propensity to crystallize, have generally been made amorphous by deliberately preventing crystallization. Recent additional preparation techniques have included like rapid precipitation by freeze drying[28] and spray drying[29] and using supercritical fluids[30], often in the presence of intrinsically amorphous hydrophilic polymers and also using methods such as melt extrusion[31]. In these systems, the drug substance is either molecularly dispersed within the polymer matrix to form a solid solution or distributed as amorphous drug domains and nanoparticulates. Under appropriate conditions of temperature and humidity, amorphous materials can crystallize when sufficient molecular mobility exists. As the amorphous phase is metastable compared to the crystalline state, there is some risk that phase transformation will occur upon storage, limiting their use in pharmaceutical dosage forms. Now a question arises; are pharmaceutical co-crystals more or less prone to polymorphism than other pharmaceutical phases?

Direct answer of this question is not possible because to prove the absence of polymorphism is tantamount to the proving-the-negative. If one considers an argument that compounds have a lower degree of self-complementarity than complementarity to a rationally selected co-crystal former. Initial indications are that polymorphic substances may provide the good candidates for the co-crystals[18]. For example; carbamazepine can exist as four different well-characterized polymorphs and a dihydrate. Co-crystals of carbamazepine and saccharin showed one packing arrangement while the co-crystals along with N,N-bis(parabromophenyl) melamine-diethyl barbital demonstrated how a specific heterosynthon between the two molecules is robust, but packing of the tapes in to a crystalline arrangement can leads to two discrete polymorphs. Hence there may be opportunity to reduce the practical extent of the polymorphism of drug compounds specifically by co-crystals formation although there may be some exception[21].

The key benefits associated with co-crystallization approach to modify properties of pharmaceutical solids are the theoretical capability of all types of drug molecules, including weakly ionizable and non-ionizable, to form co-crystals, and the existence of numerous, potential counter-molecules, including food additives, preservatives, pharmaceutical excipients as well as other APIs, for co-crystal synthesis. Major advantage is that co-crystal synthesis may offer for the pharmaceutical industry is an opportunity to address intellectual property (IP) issues by extending the life cycles of old APIs[32].

## ADVANTAGES OF CO-CRYSTALS

Co-crystals having advantages like stable crystalline form (as compared to amorphous solids), no need to make or break covalent bonds, theoretical capability of all types of API molecules (weakly ionizable/non-ionizable) to form co-crystals, the existence of numerous potential counter-molecules (food additives, preservatives, pharmaceutical excipients, and other APIs), the only solid form that is designable via crystal engineering patentable expanding IP portfolios and can be produced using solid-state synthesis green technologies high yield, no solvent or by-products.

## DESIGN OF CO-CRYSTALS

The crystal engineering experiment typically involves the Cambridge Structural Database (CSD) survey followed by the experimental work. Co-crystals designed on the principal of the supramolecular synthesis; it provides a powerful approach for proactive discovery of novel pharmaceutical solid phases. Co-crystals consist of multiple components in given stoichiometric ratio, where different molecular species interact by hydrogen bonding and by non-hydrogen bonding.

The use of hydrogen bonding rules, synthons and graph sets may assist in the design and analysis of co-crystal systems. In general though, prediction of whether co-crystallization will occur is not yet possible and must, at present, be answered empirically. Co-crystal formation may be rationalised by consideration of the hydrogen bond donors and acceptors of the materials that are to be co-crystallized and how they might interact. Following the extensive examination of preferential packing preferences and hydrogen bond patterns in a number of organic crystals, Etter and co-workers proposed the guidelines to facilitate the deliberate design of hydrogen-bonded solids[33]. All good proton donors and acceptors are used in hydrogen bonding, six-membered ring intermolecular hydrogen bonds form in preference to intermolecular hydrogen bonds, the best proton donor and acceptor remaining after intermolecular hydrogen-bond formation will form intermolecular hydrogen bonds to one another (but not all acceptors will necessarily interact with donors). These observations help to address the issue of competing hydrogen bond assemblies observed when using a particular cocrystallising agent.

A detailed understanding of the supramolecular chemistry of the functional groups present in a given molecule is the prerequisite for designing the co-crystals because it facilitates the selection of the suitable co-crystal former. Supramoecular synthons that can occur in common functional group in order to design new co-crystals and certain functional groups such as carboxylic acids, amides and alcohols are particularly amenable to formation of supramolecular heterosynthon[34]. The strong hydrogen bond includes (N-H---O), (O-H---O), (-N-H---N,) and (O-H---N). The weak hydrogen bonds involves the −C-H---O and C-H---O=C[15].

## METHODS OF PREPARATION OF CO-CRYSTALS

Co-crystal formation described in the literature indicates the notoriously difficult situation these systems present with regard to preparation it has been known to take 6 months to prepare a single co-crystal of suitable quality for single X-ray diffraction analysis[35]. This is partly because such a heteromeric system will only form if the non-covalent forces between two (or more) molecules are stronger than between the molecules in the corresponding homomeric crystals. Design strategies for co-crystal formation are still being researched and the mechanism of formation is far from being understood[36].

Co-crystals can be prepared by solvent and solid based methods. The solvent-based methods involve slurry conversion solvent evaporation, cooling crystallization and precipitation. The solid based methods involve net grinding; solvent-assisted grinding and sonication (applied to either to wet or dry solid mixtures) 80° to 85°[37].

### Solution co-crystallization:

For solution co-crystallization, the two components must have similar solubility; otherwise the least soluble component will precipitate out exclusively. However similar solubility alone will not guarantee success. It has been suggested that it may be useful to consider polymorphic compounds, which exist in more than one crystalline form as co-crystallising components. If a molecular compound exists in several polymorphic forms it has demonstrated a structural flexibility and is not locked into a single type of crystalline lattice or packing mode. Thus, the chance of bringing such a molecule into a different packing arrangement in coexistence with another molecule is increased. Clearly polymorphism alone does not guarantee the functionality of a compound to act as a co-crystallising agent, whilst the ability of a molecule to participate in intermolecular interactions obviously plays a critical role[38].

Small-scale preparation has been described. Scale-up crystallization was performed in a 500 ml water-jacketed glass crystallization vessel. Temperature was maintained by a circulating water bath. A reflux column, digital thermometer, and overhead stirrer with a glass shaft and Teflon blade were attached to vessel ports. The drug and co-crystal former were added to a reaction vessel. The solids were dissolved in ethanol/methanol mixture and heated to 70° for 1 h under reflux. Temperature was decreased in 10° increments to induce precipitation in a stirred, unseeded system. Observe the appearance of the co-crystal. Literate to enhance solids recovery decrease the further temperature[21].

### Grinding:

When preparing co-crystals, the product obtained from grinding is generally consistent with that obtained from solution. This may indicate that hydrogen-bond connectivity patterns are not idiosyncratic or determined by non-specific and unmanageable solvent effects or crystallization conditions. Nevertheless there are exceptions. Whilst many co-crystal materials can be prepared from both solution growth and solid-state grinding, some can only be obtained by solid-state grinding. An example is that in the co-crystallisation of 2,4,6-trinitrobenzoic acid and indole-3-acetic acid, different crystal forms were obtained from solution compared with grinding. Failure to form co-crystals by grinding may be due to an inability to generate suitable co-crystal arrangements rather than due to the stability of the initial phases. When co-crystal formation has been successful from solution, but not from grinding, solvent inclusion in stabilizing the supramolecular structure may be a factor. Although co-crystal formation by solid-state grinding has been established for some time and a late 19^th^ century report is often cited as the earliest reference to such a procedure, the recent technique of adding small mounts of solvent during the grinding process has been shown to enhance the kinetics and facilitate co-crystal formation and as lead to increased interest of solid-state grinding as a method for co-crystal preparation[38].

### Slurry conversion:

Slurry conversion experiments were conducted in different organic solvents and water. Solvent (100 or 200 ml) was added to the co-crystal (20 mg) and the resulting suspension was stirred at room temperature for some days. After some days, the solvent was decanted and the solid material was dried under a flow of nitrogen for 5 min. The remaining solids were then characterized using PXRD.

### Antisolvent addition:

This is one of the methods for precipitation or recrystalization of the co-crystal former and active pharmaceutical ingredient. Solvents include buffers (pH) and organic solvents. For example preparation of co-crystals of aceclofenac using chitosan, in which chitosan solution was prepared by soaking chitosan in glacial acetic acid. A weighed amount of the drug was dispersed in chitosan solution by using high dispersion homogenizer. This dispersion was added to distilled water or sodium citrate solution to precipitate chitosan on drug[39].

## INFLUENCE OF PROCESS VARIABLES ON CRYSTAL HABIT

A crystalline particle is characterized by definite external and internal structures. Habit describes the external shape of a crystal, where as polymorph state refers to the definite arrangement if molecules inside the crystal lattice[40]. Supersaturation, nucleation and crystal growth are the basic three steps in crystallization. Thermodynamic parameter like solubility, kinetical parameter like supersaturation, nucleation rate, dissolution rate, antisolvent addition rate, and evaporation rate phenomenon governs the crystallization[41].

From the above described methods such as solution crystallization, solvent change that create supersaturation by increasing the solute concentration and decreasing the solute solubility, respectively[41]. There are process variables of crystallization like, supersaturation, rate of cooling and degree of solution agitation, nature of crystallizing solvent, temperature of crystallalizing solvent and presence of impurity having possible influence on crystal habit, crystal/dosage form performance[40,42].

In case of increased saturation, rate of nuclei formation is greater than crystal growth. More growth in one direction produces fine needle shaped crystals that exhibit poor folowability, while less saturation leads to platy crystals which exhibit greater dissolution rate. Cooling a supersaturated solution of drug or pouring it into crystallizing solvent maintained at low temperature immediately decreases the drug solubility and results in rapid deposition of drug molecules on the nuclei. Rapid cooling leads to formation of platy or needle shaped crystals, slow rate of cooling forms compact, symmetric or elongated prisms. The degree of solution agitation has influence on saturation level, high speed of agitation leads to elongated crystals with small particle size distribution having good folowability and less sedimentation in suspension. Slow speed of agitation or unstirred solution forms large platy crystals.

The nature of solvent has been found to have a profound effect on crystal habit of ibuprofen. Crystallization of ibuprofen from ethanol and acetone having high surface tension, dielectric constant and less specific gravity were thin, platy, and neatly circular in shape, while those obtained from propylene glycol and 2-propanol were rod shaped. When pH was decreased by addition of hydrochloric acid to sodium hydroxide solution (pH-10) resulted in formation of needle shaped crystals. However, spherical agglomerates were obtained when ibuprofen was dissolved in acetonitrile because of limited miscibility with water[43].

Low temperature of crystallizing solvent produces irregular shaped crystals while in case of high temperature nuclei formation is delayed and fine, symmetric crystals are produced. Ions, polymeric molecules, or the other substances present in solute or solvent acts as impurities for the growing crystals and modify crystal habit. Impurity is known to modify the growing crystals into a morphology that is desirable from the viewpoint of dosage form design and performance[44]. Grinding is well known to create lattice defects and amorphous phases, and the formation of polymorphic forms of drugs as a result of these stresses is well documented in the pharmaceutical literature. Co-crystal formation during co-grinding and storage is mediated by amorphous phase, the rate of co-crystallization is dependent on the process and storage temperature, glass transition temperatures of reactants and additives, milling time and mill type[15,45].

## CASE STUDIES OF PHARMACEUTICAL CO-CRYSTALS

The earliest example of pharmaceutical co-crystals in the context of APIs relates to a series of studies conducted in the 1950s by Higuchi and Roy. They studied complex formation between macromolecules and certain pharmaceuticals. However, these would not be classified as pharmaceutical co-crystals according to the criteria applied herein[46,47].

Perhaps the first application of crystal engineering to the generation of pharmaceutical co-crystals was a series of studies reported by Zerkowski *et al.*[48] concerning the use of substituted barbituric acid, including barbital and melamine derivatives, to generate supramolecular linear tape, crinkled tape, and rosette motifs sustained by robust supramolecular synthons with three point hydrogen bonding[47]. Despite their success in cocrystal formation, the focus of these studies was not so much the physical properties of the resulting co-crystals but rather the supramolecular functionality of barbitals and their complementarities with melamine. Nevertheless, these studies illustrated very well the potential diversity of forms that can exist for a particular API as more than 60 co-crystals were structurally characterized in this series of studies. Clearly, such a diversity of forms could offer an exciting opportunity to novel and improved crystalline forms of APIs. Herein, we have chosen to focus upon several case studies that involve the formation of pharmaceutical co-crystals with altered physical properties of clinical relevance.

### Pharmaceutical co-crystals of carbamazepine (Tegretol®):

Carbamazepine (CBZ) is an important antiepileptic drug that has been in use for over three decades. Oral administration of CBZ encounters multiple challenges, including low water solubility with high dosage required for therapeutic effect (i.e. >100 mg/day), dissolution-limited bioavailability and auto induction for metabolism. In contrast to its simple molecular structure, CBZ exhibits complexity in its crystal forms[20,21]. To date, four anhydrous polymorphs, a dihydrate, an acetone solvate, and two ammonium salts of CBZ have been identified. It is noted that, in the crystal structures of all these forms, the self-complementary nature of the amide group manifests itself in a predictable manner. Therefore, CBZ has been used as an ideal candidate to demonstrate how APIs can be converted to pharmaceutical co-crystals, and how these co-crystals could offer optimized physicochemical properties over existing forms of an API[20,43]. Two strategies have been adopted for co-crystal formation of CBZ. One crystal engineering strategy is to employ the peripheral hydrogen bonding capabilities that are not engaged in the pure form of CBZ. A second strategy for co-crystallization of CBZ involves breakage of the CBZ amide-amide dimer and formation of a supramolecular heterosynthon between CBZ and a co-crystal former[43]. Both strategies are successful and have afforded a number of CBZ co-crystals that exhibit improved physicochemical properties. For example, the CBZ:saccharin co-crystal shows significantly improved physical stability (i.e. only one cocrystal form with equivalent chemical stability to the anhydrous polymorph has been identified after sophisticated form screening)[21]. In addition, the CBZ:saccharin cocrystal possesses favorable dissolution properties, suspension stability, and pharmacokinetics using dog models. The pharmacokinetic study reveals that the CBZ:saccharin co-crystal exhibits a higher C_max_ and comparable T_max_ when compared with the marketed form, Tegretol®[21]. In short, the CBZ:saccharin cocrystal appears to be superior to existing crystal forms of CBZ in the following respects: stability relative to the anhydrous polymorph of CBZ; favorable dissolution and suspension stability, favorable oral absorption profile in dogs[49].

### Pharmaceutical co-crystals of fluoxetine hydrochloride (Prozac®):

The availability and marketability of a variety of APIs as chloride salts is well recognized, and, recently, an approach to utilize such chloride salts, specifically fluoxetine hydrochloride (fluoxetine HCl), to generate co-crystals of an amine hydrochloride salt via a chloride-mediated carboxylic acid supramolecular synthon has been reported. Fluoxetine HCl is the active pharmaceutical ingredient found in the common antidepressant drug Prozac®. It is a solid under ambient conditions, only one crystalline phase is known, and it is available in the salt form. It has been demonstrated that co-crystallization of this API modifies the physical properties of fluoxetine HCl while still retaining the hydrochloride salt of the API. Fluoxetine HCl was co-crystallized with benzoic acid (1:1), succinic acid (2:1), and fumaric acid (2:1) via traditional evaporation techniques. For all three co-crystals, the carboxylic acid was found to form hydrogen bond to the chloride ion, which in turn interacted with the protonated amine, thus generating, in all three cases, amine hydrochloride salt hydrogen bonding to an additional neutral molecule. Powder dissolution experiments were carried out in water for the three novel co-crystals resulting in a spread of dissolution profiles. The fluoxetine HCl:benzoic acid cocrystal was found to have a decrease in aqueous solubility by 50%, and the fluoxetine HCl:fumaric acid cocrystal had only a slight increase in aqueous solubility. However, the fluoxetine HCl: succinic acid cocrystal exhibited an approximately twofold increase in aqueous solubility after only 5 min. The complex formed between succinic acid and fluoxetine HCl falls apart in solution to generate its pure components after about 1 h. An intriguing aspect of this study is that by simply hydrogen bonding a hydrochloride salt of an API with similar cocrystal formers, one can generate distinctively different dissolution profiles[50].

### Pharmaceutical co-crystals of itraconazole (Sporanox®):

Itraconazole is a triazole antifungal agent that is prescribed to patients with fungal infections. Itraconazole is extremely water insoluble and administered both orally and intravenously. The oral formulation of itraconazole is the amorphous form coated on the surfaces of sucrose beads, and marketed as the Sporanox® capsule. In addition, co-administration of acidified HP-β-cyclodextrin beverages with Sporanox® capsules is required to achieve the maximal absorption of the API, even though such a co-administration can cause diarrhea[51,52]. Interestingly, no crystalline salt of itraconazole has been reported in the patent literature, even though salt formation using itraconazole and an acidic salt former would seem to be a logical approach to improve the absorption properties of the API. In order to improve the absorption of the API and maintain the form crystallinity and stability, the pharmaceutical cocrystal approach has been evaluated in the formulation of itraconazole.

Crystalline phases of itraconazole can be engineered by introduction of additional molecules to match hydrogen-bond donors and acceptors[49,50]. A number of stable pharmaceutical co-crystals of itraconazole and 1,4-dicarboxylic acids were synthesized and crystallographically characterized[45]. Each cocrystal contains two API molecules and one acid cocrystal former, hydrogen- bonded through carboxylic acid–triazole supramolecular synthons, to form a trimeric assembly. The aqueous dissolution of itraconazole co-crystals was studied in order to assess their potential impact on bioavailability of the API. The dissolution of itraconazole co-crystals was observed to behave more akin to the Sporanox form than to the crystalline form of the pure API. In particular, it was noted that the itraconazole:L-malic acid cocrystal exhibits a similar dissolution profile to that of the marketed formulation[49]. In a further pharmacokinetic study of itraconazole co-crystals, it was revealed that cocrystal formulation of the API gives similar oral bioavailability to the Sporanox form in the animal trial using a dog model[50]. In short; this study demonstrates the use of pharmaceutical co-crystals for the improvement of solubility and bioavailability without compromising crystallinity and stability.

### Pharmaceutical co-crystals of sildenafil (Viagra®)[53]:

Sildenafil is a drug used in the treatment of pulmonary arterial hypertension, congestive heart failure, atherosclerosis, conditions of reduced blood vessel potency and peripheral vascular disease, as well as male erectile dysfunction and female sexual disorders. Sildenafil selectively inhibits cyclic guanosine monophosphate (cGMP)-specific phosphodiesterase type 5 that is responsible for degradation of cGMP in the corpus cavernosum, leading to smooth muscle relaxation in the corpus cavernosum, and resulting in increased inflow of blood and an erection. Sildenafil citrate, with moderate water solubility, has been commercially developed and marketed by Pfizer and is available under the trademark Viagra®.

It has been observed that sildenafil in a pharmaceutical cocrystal form could provide an improved solubility of the API under acidic conditions. In addition, such an improvement of solubility of sildenafil could be particularly advantageous for its orally administrable formulation. Sildenafil has been successfully co-crystallized with acetylsalicylic acid (1:1 molar ratio) by slurry or under reflux conditions.

The crystal structure of the cocrystal of sildenafil and acetylsalicylic acid has been determined by single crystal X-ray diffraction, and in addition, the composition of matter was confirmed by powder X-ray diffraction and infrared spectrometry. Moreover, the differential scanning calorimetry and thermo gravimetric analyses indicate that the melting point of the cocrystal is approximately 143°, and it remains thermodynamically stable up to 165°[31]. An intrinsic dissolution study in simulated gastric body fluid (pH 1.2) shows that the sildenafil: acetylsalicylic acid cocrystal exhibits an intrinsic dissolution rate (IDR) of ca. 11.75 mg/min/cm vs. 6.64 mg/min/cm for sildenafil citrate under the same conditions.

### Co-crystal of melamine and cyanuric acid:

In early 2007, the FDA received complaints from owners of more than 4000 pets regarding the deaths of animals after taking food that was later recalled; it was reported that majority of those deadly incidents were caused by acute renal failure. At first, melamine, which was observed in the tainted products, was the suspected contaminant, since this particular chemical could be intentionally added to raise the apparent protein content of the food. However, melamine is considered relatively nontoxic with the acute toxicity of melamine in rats was reported with oral lethal doses 50 (LD_50_) of 3100 mg/kg for the male and 3900 mg/kg for the female. Also, the quantity of melamine observed in those incidents was not at levels that would normally kill. In the course of the pet food recall investigation, cyanuric acid, another relatively nontoxic compound, was also identified in the pet food as a co contaminant. Although melamine and cyanuric acid are relatively safe individually, no data could be found in the literature that could determine the potential toxicity of melamine and cyanuric acid in combination. From the crystal engineering viewpoint, melamine and cyanuric acid (1:1 molar ratio) form an extensive two-dimensional networks in the solid-state based on the robust three-point molecular recognition, and it was observed that the resulting melamine:cyanuric acid cocrystal is highly insoluble in water.

As reported by a recent investigation, the combination of melamine and cyanuric acid can result in the intra tubular precipitation of melamine:cyanuric acid co-crystals in the kidney, even though the mechanism associated with renal damage is not fully understood to date. A study conducted at the Bergh Memorial Animal Hospital in New York revealed that co-crystals blocked the tubes leading from the kidneys to the bladder in one cat, and a toxicology assessment of melamine and cyanuric acid indicated that a single oral exposure of cats to the melamine:cyanuric acid cocrystal at a concentration of 32 mg/kg body weight can result in acute renal failure. It seems clear that the formation of a low solubility cocrystal of melamine and cyanuric acid is responsible for these incidents. Perhaps this case study of melamine:cyanuric acid co-crystals are the first example showing how co-crystals can significantly alter the relevant physical properties in a negative manner[54].

### Co-crystals of theophylline:

Theophylline is useful in treatment of respiratory disease such as asthma. From the physicochemical standpoint, theophylline represents challenge to formulators in that it is known to interconvert between crystalline anhydrate and monohydrate forms as a function of relative humidity (RH). The possibility of crystalline hydrate formation complicates design of a consistent, reproducible for an API in the drug development process. Reversible hydrate formation is particularly problematic, as it indicates that neither the anhydrate nor the hydrate is fully stable across the range of common processing condition. Theophylline is structural analogue of caffeine. The co-crystals of the theophylline were prepared with oxalic acid, malonic acid, maleic acid, glutaric acid by solvent evaporation technique. The relative humidity stability comprised of the storage and subsequent PXRD analysis at four specific RH levels (0%, 43%, 75% and 98% RH) across four different time points (1 day, 3 day, 1 and 7 weeks). Over the course of 7 week study it was found that, at 75% RH and below, theophylline anhydrate converted into theophylline monohydrate. No formation of theophylline hydrate was found in any case.

The observed RH stability of theophylline co-crystal demonstrates the physical stability improvement, specifically avoidance of hydrate formation. The co-crystals formed by oxalic acid found to be more stable. This study demonstrates use of co-crystals in physical property improvement[17].

### Co-crystals of aceclofenac:

Aceclofenac is an orally effective nonsteroidal antiinflammatory drug of phenyl acetic acid group, which possesses remarkable antiinflammatory, analgesic and antipyretic properties. Aceclofenac exhibits slight solubility in water and as a consequence it exhibits low bioavailability after oral administration. Mutalic[39] prepared co-crystals of aceclofenac by simple solvent change approach by using chitosan.

Chitosan has been considered to be one of the most promising biopolymer for drug delivery purpose. Chitosan is a linear hydrophilic polysaccharide polymer of D-glucosamine. It is non-toxic natural polcationic polymer that is degraded by the microflora in the colon. It is abundant in nature and is present in the exoskeleton of crustaceans such as crabs and shrimps. Chitosan has been demonstrated to be a good vehicle for enhancing the dissolution properties and bioavailability of a number of poorly water-soluble drugs[55].

Chitosan was precipitated on aceclofenac crystals using sodium citrate as a salting out agent. The pure drug and prepared co-crystals with different concentrations (0.05 to 0.6%) were characterized in terms of solubility, drug content, particle size, thermal behavior (differential scanning calorimetry, DSC), X-ray diffraction (X-RD), morphology, *in vitro* drug release, stability and phrmacokinetic study.

It was observed that particle size of co-crystals was drastically reduced during the formulation process. The DSC showed a decrease in melting enthalpy indicating disorder in crystallinity. XRD also revealed disorder in crystallinity. The dissolution study showed that marked increase in dissolution rate in comparison to pure drug. The considerable dissolution rate of aceclofenac from optimized crystal formulation was attributed to the wetting effect of chitosan, decreased drug crystallinity, altered morphology and micronization. The optimized crystals showed excellent stability on storage at accelerated conditions. *In vivo* study revealed that the crystals provided a rapid pharmacological response in mice and rat; besides improve in pharmacokinetic parameters in rats[39].

### Co-crystal of 5-nitrouracil:

Co-crystals of 5-nitrouracil with solvent molecules, dioxane, pyridine, DMSO, formamide and ethanol as well as with piperazine, N,N'-dimethylpiperazine, 3-aminopyridine and diazabicyclooctane obtained by deliberate inclusion, have been examined by X-ray crystallography. The tape structure found in the parent centric form of nitrouracil is retained with some modifications in the co-crystals with dioxane, piperazine, diazabicyclo-octane, N,N'-dimethylpiperazine, pyridine and DMSO, with the guest molecules forming alternate tapes. In co-crystals involving formamide, ethanol and 3-aminopyridine, the molecular tapes exhibit mixed compositions. The observed bonding patterns have been classified into six schemes. Interestingly, quadruple type hydrogen bonding patterns are seen in co-crystals containing 3-aminopyridine or ethanol and water, while a network of acyclic tetrahedral pentamers of water is found in the cocrystal containing diazabicyclo-octane and water. This case study reveals that hydrogen acceptors and donors are necessary to form cocrystal[56].

### Co-crystals of indomethacin:

Indomethacin, a non-steroidal antiinflammatory drug (NSAID), is widely prescribed for patients with arthritis. It exists as α, γ and amorphous forms, in the solid state α-form being most stable form at room temperature. Indomethacin is practically insoluble in water (2.5-4 μg/ml; it belongs to BCS class II) and poses severe challenges in the formulation development. Various cocrystal formers, including saccharin, were used in the screening for indomethacin co-crystals in a series of solvents.

Solution evaporation method was used in the screening phase. DSC, TGA, IR, Raman and PXRD techniques characterized the potential new phases. The indomethacin saccharin co-crystal (IND-SAC co-crystal) structure was determined from single crystal X-ray diffraction data. Pharmaceutically relevant properties such as the dissolution rate and dynamic vapour sorption (DVS) of the IND-SAC co-crystal were evaluated. Solid-state and solvent-drop co-grinding methods were also applied to indomethacin and saccharin.

The IND-SAC co-crystals were obtained from ethyl acetate. Physical characterization showed that the IND-SAC co-crystal is unique vis-à-vis thermal, spectroscopic and X-ray diffraction properties. The co-crystals were obtained in a 1:1 ratio with a carboxylic acid and imide dimer synthons. The dissolution rate of IND-SAC was considerably faster than that of the stable indomethacin α-form. DVS studies indicated that the co-crystals gained less than 0.05% in weight at 98% RH. IND-SAC co-crystals were also obtained by solid state and solvent-drop co grinding methods. The IND-SAC co-crystal was formed with a unique and interesting carboxylic acid and imide dimer synthons interconnected by weak N-H…O hydrogen bonds. The co-crystals were associated with significantly faster dissolution rate than indomethacin (α-form) in phosphate buffer pH 7.4 and were non-hygroscopic[57].

## CO-CRYSTALS AND PATENTS

“New” refers to anything under the sun that is made by the man, such as new composition of matter or any useful improvements. Thus a necessary condition to claim a new composition of matter is to describe clearly with precision the composition, a great challenge in the case of co-crystals[58].

Aripiprazole co-crystals[59], the present invention relates to co-crystals comprising aripiprazole and fumaric acid and processes for co-crystal preparation. Aripiprazole is a psychotropic drug useful for the treatment of schizophrenia and is the sixth, and most recent, of the second generation antipsychotic medications. It is available in the market under the brand name Abilify® in the form of tablets of 5, 10, 15, 20 and 30 mg strengths. Aripiprazole presents certain challenges for formulation as a rapid-onset dosage form, particularly as a rapid-onset oral dosage form. For example, aripiprazole has a very low solubility in aqueous media (being practically insoluble) and therefore is not readily dissolved and dispersed for rapid absorption in the gastrointestinal tract when administered orally, for example in tablet form. Towards this end, it has been the endeavor of pharmaceutical scientists to provide new forms of aripiprazole, more specifically, a thermodynamically stable form which would have the strengths of the crystalline forms, viz. thermodynamic stability, and those of the amorphous form, viz. enhanced solubility, rapid onset of action and an enhanced bioavailability.

Consequently, there is a need for soluble forms of aripiprazole that can be readily formulated for use in various modes of administration, including parenteral and oral administration. Co-crystal complexes of aripiprazole would add a powerful tool in the treatment of central nervous system disorders. The present invention provided co-crystals of aripiprazole and fumaric acid which are stable and are reproducible on an industrial scale.

TDFA is the co-crystal of tenofovir disoproxil hemi-fumaric Acid[60]. Tenofovir disoproxil fumarate (DF) is a nucleotide reverse transcriptase inhibitor approved in the United States for the treatment of HIV-1 infection alone or in combination with other antiretroviral agents. Tenofovir disoproxil DF is sold under the trade name Viread (Gilead Science, Inc.) and present in combination with other antiviral agents in the Truvada and Atripla. After analysis of several commercially available products containing tenofovir DF, it was found that these contained mixtures of solid forms of tenofovir DF in varying ratios. Indications have been found by the present inventors that the solid form of tenofovir DF in commercially available products is generally a mixture of at least two forms. It has also been found that one of these forms experiences a conversion of its crystalline form into the other form when put under stress, such as increased temperature and/or humidity. It is believed by the present inventors that the presence of water will induce or enhance the conversion of one form into the other. This suggests that the solid form currently used in the marketed product is not stable or at least has a reduced stability. The bulk molar ratio of tenofovir disoproxil to fumaric acid in the commercially available products is generally indicated as 1:1. The TDFA 2:1 co-crystal of the invention is more stable and is less hygroscopic than the presently known crystalline form of tenofovir.

Theophylline crystallized rapidly from a hot ethylene glycol solution forms theophylline co-crystals[61]. This technique was confirmed as being functional by testing for a known co-crystal of theophylline and p-nitrophenol. Salicylic acid, p-hydroxybenzoic acid, sorbic acid, 1-hydroxy-2-naphthoic acid, glycolic acid, and 2,5-dihydroxybenzoic acid were all tested as guest compounds and in each, a co-crystal formation had occurred. Raman spectra of the pure guest acid, theophylline, and the co-crystal were obtained and compared to confirm co-crystal formation. This invention provides information regarding a new method for co-crystallization i.e ethylene glycol based method.

As one aspect, novel co-crystals are provided. The novel co-crystals comprise one or more active agents, particularly of the salts of such active agents. Novel forms of salts of active pharmaceutical ingredients are provided. For example, the present invention provides novel co-crystals of fluoxetine HCl and benzoic acid; fluoxetine HCl and succinic acid; and fluoxetine HCl and fumaric acid. Novel forms or solid state phases of active pharmaceutical ingredients may be prepared for which there are no known polymorphs, solvates or hydrates, or where such polymorphs, solvates or hydrates were disfavored[62]. Co-crystals fulfill the criteria for patent eligibility: novelty, utility, and non obviousness.
